# Reversible craniocervical dystonia associated with levofloxacin

**DOI:** 10.1186/s40734-015-0021-8

**Published:** 2015-05-04

**Authors:** Karlo J Lizarraga, Maria R Lopez, Carlos Singer

**Affiliations:** Department of Neurology, University of Miami, Miller School of Medicine, Clinical Research Building, 1120 NW 14th Street, 13th Floor, Miami, FL 33136 USA

**Keywords:** Craniocervical dystonia, Levofloxacin, Quinolones, Renal disease

## Abstract

**Background:**

Dystonia is a hyperkinetic movement disorder related to dysfunction of inhibitory basal ganglia and cortical circuits. GABA is the major inhibitory neurotransmitter in the central nervous system. Quinolones can rarely produce serious neurologic effects, which have been attributed to impaired inhibition through GABA antagonism. We report a case of reversible craniocervical dystonia associated with oral levofloxacin.

**Case presentation:**

A 62-year-old man on hemodialysis presented with craniocervical dystonia 3 days after initiation of levofloxacin 500 mg. twice daily. Levofloxacin was discontinued. Seven days later the abnormal movements completely disappeared and did not recur in the following 3 months.

**Conclusion:**

Levofloxacin should be considered as a rare but potentially reversible trigger of craniocervical dystonia. Older age, renal impairment and high doses of the drug might be risk factors.

**Electronic supplementary material:**

The online version of this article (doi:10.1186/s40734-015-0021-8) contains supplementary material, which is available to authorized users.

## Background

Dystonia is a hyperkinetic movement disorder characterized by typically patterned, sustained or intermittent muscle contractions producing abnormal, often repetitive, movements, postures, or both. It is often precipitated or worsened by voluntary action and associated with overflow muscle activation [1]. Defective inhibition in basal ganglia and cortical pathways are thought to play a significant role in the pathophysiology of dystonia [2].

Quinolones have γ-aminobutyric acid (GABA) type-A receptor antagonist properties [3], and can thus impair inhibition and act as excitatory compounds in central nervous system (CNS) circuits. They have been rarely reported to cause abnormal hyperkinetic movement disorders. These reactions have been particularly described in patients taking ciprofloxacin, the quinolone with the most potent GABA antagonist effect [3,4].

We report a case of reversible craniocervical dystonia associated with oral levofloxacin therapy in an end-stage renal disease (ESRD) patient on hemodialysis (HD).

## Case presentation

A 62-year-old, left-handed, Caucasian man undergoing HD every-other-day due to diabetes mellitus related ESRD, presented with intermittent episodes of involuntary blinking, speech stuttering and neck flexion on attempted oral communication. These episodes were particularly severe with stress or anxiety. Patient denied a preceding urge to move or talk. Patient denied oscillopsia, diplopia, ear clicking, tinnitus, dysphagia or hallucinations. There was no history of prior exposure to dopamine-blocking medications or quinolones. Patient was compliant with multiple medications for diabetes mellitus and ESRD. Three days prior to the onset of abnormal movements, patient had been empirically prescribed levofloxacin 500 mg. orally twice daily for urinary symptoms. No other medication had been recently added or discontinued, and there was no recent dose change on his usual medications. There was no family history of abnormal movements.

On examination, the patient was alert and fully oriented. Dystonic movements of upper facial, oromandibular and cervical muscles causing involuntary blinking, blepharospasm, tongue tremor and protrusion, dysarthria, platysmata contractions and anterocollis were observed. Bilateral ocular and palatal movements were normal without motor impersistence on voluntary tongue protrusion [Additional file 1: Video 1]. There was no facial or cervical muscle weakness. There were no other associated abnormal movements including limb dystonia, chorea or parkinsonism. There were no upper motor neuron or cerebellar signs.

Laboratory studies disclosed stable values in complete blood count and comprehensive metabolic panel including electrolytes and renal function tests (creatinine level range: 3.8–5.1 mg/dL). Urine culture was negative. No imaging studies were obtained.

Levofloxacin was discontinued and patient noted progressive improvement in the duration and severity of the dystonic episodes. Seven days later they had completely disappeared [Additional file 2: Video 2]. Furthermore, there were no more episodes in the following 3 months.

## Discussion

According to recent guidelines, we use the term craniocervical dystonia instead of the eponym “Meige syndrome” to describe this patient’s intermittent episodes of involuntary blinking, blepharospasm, tongue protrusion, platysmata contractions and anterocollis ([1,5], Additional file 1: Video 1). Most of these movements were observed when he attempted to talk and were not preceded by an intense urge to move or talk. Tongue tremor at rest and patterned episodes of tongue protrusion while maintaining an open mouth were noted [Additional file 1: Video 1]. In fact, dystonic tremor has been described as inconstant and often exacerbated by an attempt to maintain posture [6]. Finally, this patient did not complain of dysphagia and there were no abnormal movements of the jaw.

In patients with craniocervical dystonia, an 11% chance of spontaneous remission has been reported, particularly those with blepharospasm [7]. Yet, it is important to identify potentially reversible causes.

Fluoroquinolones are commonly used antibiotics that can have CNS excitatory effects through a dose-dependent, GABA-A receptor antagonist mechanism [3,4]. Ciprofloxacin has been shown to be a more potent GABA antagonist than other quinolones *in vitro* [3]. Moreover, an *in vivo* EEG study demonstrated pronounced decrease in delta and theta, as well as augmentation of alpha wave bands after ofloxacin infusion. These effects were augmented by flumazenil and reversed by midazolam [4]. In general, quinolones are rarely associated with neurological adverse reactions, mostly dizziness, headache and insomnia. They have a <0.5% rate of serious neurological reactions including abnormal movements, alteration of consciousness and epileptic spells. However, ciprofloxacin has a rate of up to 4% [8-10]. New agents are developed by chemically modifying older compounds to improve their spectrum, potency, pharmacokinetics and safety. Levofloxacin, derived from ofloxacin, received marketing approval in the US in 1997. Remarkably, the recommended dose of levofloxacin is equivalent to a 2.5 times higher ofloxacin dose. Levofloxacin has a <0.001% rate of severe neurologic reactions, mostly reported as convulsions [8-10].

A diverse reversible hyperkinetic movement phenomenology has been reported in relation to ciprofloxacin (tremor [9,11], myoclonus [12-14], orofacial dyskinesias [15,16], chorea [17], tic disorder [18], palatal tremor [19] and stereotypy [17]), levofloxacin (tremor and chorea [20]), ofloxacin (tic disorder [21]) and gemifloxacin (dystonia [22]).

The pathophysiology of dystonia encompasses dysfunction of inhibitory basal ganglia and cortical circuits [2]. Through GABA antagonism, quinolones could potentially predispose to abnormal inhibition and trigger dystonic movements. To the best of our knowledge, dystonia has not been reported in association with levofloxacin. Additionally, segmental dystonia has not been described as a side effect of quinolones.

A case of acute-onset multifocal dystonia (involving the four extremities with sparing of axial muscles) has been reported in a 36 year-old woman three days after initiation of gemifloxacin. Her abnormal movements quickly resolved with intravenous administration of promethazine 50 mg [22]. A 43 year-old woman presented with confusional state, “dystonic posturing of the feet”, diffuse chorea and stereotypy preceding generalized tonic-clonic seizures after ciprofloxacin intake [17]. In another report, a 49 year-old woman was noted to have intermittent 1-hour episodes of facial grimacing every 4–6 hours that were described as “oral facial dyskinesia” induced by ciprofloxacin [15], but may have included blepharospasm. Finally, a 68 year-old man taking ciprofloxacin was noted to have 15–30 second episodes of “orofacial dyskinesias” consisting of facial grimacing and distortions, puckering and pursing of the lips, without eyelid involvement. These episodes resolved shortly after ciprofloxacin was discontinued [16].

Levofloxacin is excreted primarily unchanged in urine [23]. Therefore, dose adjustments are required in individuals with impaired renal function. HD does not effectively remove the drug from the body [24]. Dosing guidelines recommend 500 mg on the first day, followed by 250 mg every 48 hours for patients with ESRD on HD [25]. Our patient, an older ESRD patient on HD, was taking 500 mg twice daily for 3 days before dystonia onset.

As hypothesized in other reports, neurotoxic levels of levofloxacin were likely reached due to a combination of renal impairment, age-related decline in cerebral function and reduction of plasma volume leading to higher plasma and CNS drug concentrations. Similar to other reported cases of quinolone-associated hyperkinetic movements, dystonia disappeared completely after discontinuation of levofloxacin [Additional file 2: Video 2]. Benzodiazepines were not used in this case, but can be considered to reverse the GABA inhibition related to quinolone intoxication [26]. Due to the ethical implications of a “quinolone challenge”, it was felt that the time course of the patient’s dystonia in relation to levofloxacin intake and discontinuation was a strong evidence for this association.

## Conclusion

Levofloxacin should be considered as a rare but potentially reversible cause of new-onset craniocervical dystonia, particularly in older adults with renal impairment. High doses, older age and renal impairment might be risk factors for this side effect.

## Consent

Written informed consent was obtained from the patient for publication of this case report and any accompanying images or videos. A copy of the written consent is available for review by the Editor-in-Chief of this journal.

## Additional files

Additional file 1:
**Video 1.** Involuntary movements consistent with craniocervical dystonia three days after initiation of levofloxacin. Additional file 2:
**Video 2.** Resolution of craniocervical dystonia seven days after discontinuation of levofloxacin.

## Abbreviations

CNS: Central nervous system
ESRD: End-stage renal disease
GABA: γ-aminobutyric acid
HD: Hemodialysis
